# The Spectrum of Acute Disseminated Encephalomyelitis and Mild Encephalopathy with Reversible Splenial Lesion

**DOI:** 10.1155/2019/9272074

**Published:** 2019-10-17

**Authors:** Michel Sáenz-Farret, Mariana Aurora Cansino-Torres, Valeria Sandoval-Rodríguez, Rigoberto Navarro-Ibarra, Carlos Zúñiga-Ramírez

**Affiliations:** Movement Disorders and Neurodegenerative Diseases Unit, Hospital Civil de Guadalajara “Fray Antonio Alcalde”, Guadalajara, Mexico

## Abstract

**Background:**

Acute disseminated encephalomyelitis and mild encephalopathy with reversible splenial lesion are autoimmune demyelinating disorders of central nervous system. Diagnosis remains clinical, aided by neuroimaging confirmation and excluding other causes. In the absence of a biological marker, the diagnosis of these entities based on clinical and imaging criteria could overlap.

**Methods:**

We describe a 22-year-old woman developing mild neurological signs after an upper tract infection, a brain magnetic resonance image revealed confluent, symmetrical white matter lesions with corpus callosum involvement; after extensive ancillary testing that ruled out secondary causes we concluded that this subject had a post infectious encephalitis sharing clinical and imaging criteria for acute disseminated encephalomyelitis. However, mild encephalopathy with reversible splenial lesion could be an alternate diagnosis for this subject. Treatment with methylprednisolone completely solved both the clinical and image abnormalities without relapsing for more than 3 years of follow-up.

**Conclusion:**

Both acute disseminated encephalomyelitis and mild encephalopathy with reversible splenial lesion share clinical and radiological features. A biological marker is needed to differentiate among these entities, since overlap is seen according to current criteria.

## 1. Introduction

Acute disseminated encephalomyelitis (ADEM) is a multifocal inflammatory demyelinating disease of the central nervous system (CNS) which occurs most commonly in children after a bacterial, viral process or vaccination. It is thought that an immune response against these infectious agents cross-react with self-myelin peptides, hence leading to an autoimmune response [1].

In most cases the disease has a monophasic course, although it can also present as a relapsing illness. ADEM usually begins from 2 days to 4 weeks after infection or vaccination. The typical course begins with a sudden onset of encephalopathy and several focal neurological deficits such as weakness of extremities, ataxia, cranial nerve palsies, seizures, myelitis and optic neuritis among others. Subjects also develop systemic symptoms such as headache, fever and vomiting [2].

Magnetic resonance imaging (MRI) of the brain and spine is the most useful ancillary test for acute demyelination [3]. The newest proposed criteria for ADEM diagnosis based on MRI include: 1. Diffuse, poorly demarcated, large (>1–2 cm) lesions involving predominantly the cerebral white matter, 2. White matter T1 hypointense lesions are rare, and 3. Deep gray matter lesions (e.g. thalamus or basal ganglia) can be present. Furthermore, the MRI during the acute phase should be abnormal and new lesions should not be present three months or more after the disease onset [4].

An important differential diagnosis of ADEM is mild encephalopathy with reversible splenial lesion (MERS), this entity is a clinico-radiological syndrome characterized by a transient mild encephalopathy and a reversible lesion in the splenium of the corpus callosum on MRI. This syndrome has almost universally been described in children from Japan and East Asia [5]. The aim of this article is to present a case of an adult fulfilling both clinical criteria for ADEM and MERS type 2. Based on these findings, both entities could be, at least, part of the spectrum of one disease. A biological marker capable to distinguish ADEM from MERS is needed.

## 2. Case Presentation

**Figure 1:**
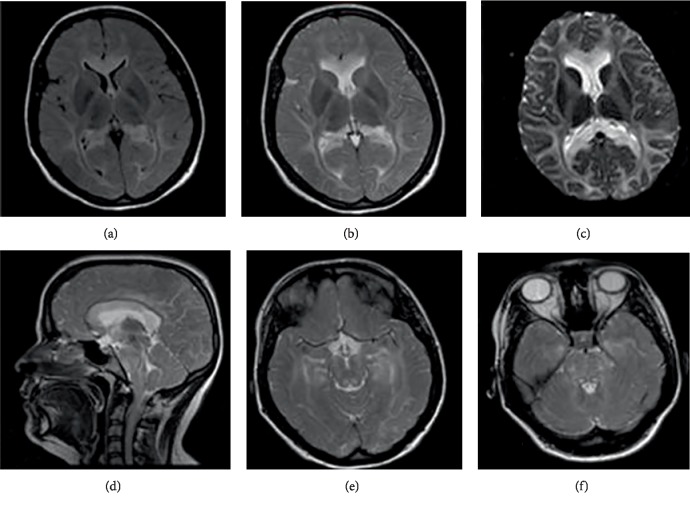
(a) Axial FLAIR showed hyperintense signal in supratentorial white matter. (b) Axial T2 showed hyperintense signal in supratentorial white matter. (c) Axial Diffusion showed restricted diffusion at the entire corpus callosum and at the white matter. (d) Sagittal T2 showed hyperintense signal in supratentorial white matter and also in the brainstem. (e) Axial T2 showed hyperintense signal at the mesencephalon. (f) Axial T2 showed hyperintense signal at the pons.

**Figure 2:**
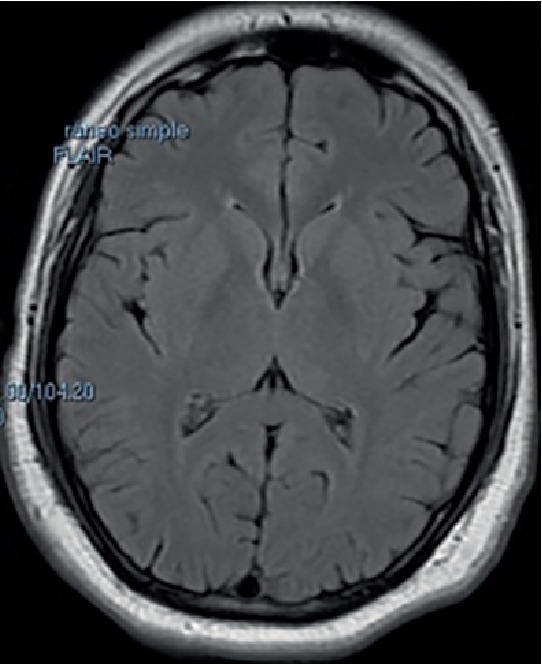
Axial FLAIR showed absence of abnormalities at follow up.

A 22-year-old woman was admitted into the hospital for a 2-week course of diplopia, frontal headache and retro-ocular pain following an unspecified self-limited upper respiratory tract infection 15 day prior to the appearance of these neurological symptoms. Neurologic exam showed conjugated primary-gaze diplopia and generalized hyperreflexia. Fundoscopy showed optic nerve pallor bilaterally. Brain MRI was performed which showed extensive T2 and FLAIR white matter hyperintense lesions as well as in brainstem and splenium of corpus callosum (Figure 1). Laboratory tests including blood count, glucose, urea, creatinine, electrolytes and HIV ELISA were unremarkable. Cerebrospinal fluid was completely normal, oligoclonal bands were absent and Polymerase Chain Reaction (PCR) for Herpes Virus Simplex Type I (HSV-I) and other common viruses was performed, resulting negative as well. Somatosensory Evoked Potentials depicted only a delay at P37 wave bilaterally. Symptoms completely solved after 5 days of intravenous methylprednisolone 1 g daily. Six-weeks after treatment, a brain MRI was repeated, showing complete resolution of previous findings (Figure 2). Complete remission was achieved clinically and radiologically, remaining asymptomatic for more than three years of follow-up.

## 3. Discussion

ADEM in adults is a rare manifestation of post infectious disease [6], however it can also arise spontaneously or after vaccination [7]. Nevertheless, among these subjects, an infectious background should raise suspicion of an autoimmune demyelinating disease.

The diagnosis of ADEM remains clinical, aided by neuroimaging confirmation and the exclusion of other causes [8]. Clinical features are varied and depend mainly on the affected CNS area. In the largest prospective study of ADEM in adults, the most frequent signs of involvement were at the brainstem, mainly ocular motor deficit followed by dysarthria. It is important to note that in contrast to children, the prevalence of fever, loss of consciousness, and Meningism was low [9] as seen in our case. Brain and spine MRI is the most useful paraclinical tool to aid in the diagnosis of ADEM, distinguishing it from other inflammatory and noninflammatory neurological diseases [10].

Five patterns of cerebral involvement have been proposed to classify the MRI findings in ADEM among children and adolescents: 1. ADEM with small lesions (less than 5 mm); 2. ADEM with large, confluent, or tumefactive lesions; 3. ADEM with additional symmetrical bithalamic involvement; 4. Acute hemorrhagic encephalomyelitis (AHEM); and 5. ADEM with pseudo-leukodystrophic pattern, with a diffuse, bilateral, symmetrical, and usually non-enhanced white matter involvement [2].

The diffuse, symmetrical, and usually non-enhancing white matter involvement can be rarely found in subjects with ADEM, with all the reported cases seen during infancy and adolescence. Clinically, most of them were associated with seizures, fever and aggressive neurologic involvement. Although these findings in neuroimaging are not the most typical pattern of ADEM, the exclusion of other entities such as a history of previous infection or vaccination points toward a diagnosis of ADEM. Also, the response to methylprednisolone supports the diagnosis in a retrospective manner [11–13].

Since ADEM is a diagnosis of exclusion [7], other diseases were considered prior to diagnosing this entity in our patient. In adults, the differential diagnosis of confluent, symmetrical white matter lesions and corpus callosum involvement is broader including inflammatory conditions, infections, vascular disorders, neoplasia, and toxic causes [14]. CNS lymphoma is an entity that must be considered among subjects with large and atypical white matter lesions. Steroids can vanish white matter abnormalities in these cases, but recurrence is always seen [15]. Our case remained free from lesions after more than 3 years of follow up, making CNS lymphoma diagnosis unlikely.

Clinical clues in the present case favoring a diagnosis of ADEM are: prior upper tract infection, unremarkable laboratory studies, confluent symmetrical white matter lesions, the complete response to methylprednisolone and the monophasic course, remaining symptomatically free for more than 3 years of follow up. Nevertheless, atypical MRI findings such as corpus callosum involvement, a confluent, symmetrical white matter lesions and subtle neurological involvement makes also MERS type 2 a feasible diagnosis.

MERS is a clinical and radiological syndrome characterized by transient lesions at the splenium and associated frontal white matter [16]. The diagnostic criteria for MERS includes: onset with neuropsychiatric symptoms within one week after the onset of fever; complete recovery without sequelae within 10 days after the onset of neuropsychiatric symptoms; high-signal-intensity lesion in the splenium of corpus callosum, in the acute stage; lesion may involve the entire corpus callosum and the cerebral white matter in a symmetric fashion; lesion disappears within 1 week, with neither residual signal changes nor atrophy [17]. Additionally, parietal or frontoparietal lesions have also been reported in MERS [18] but not confluent, symmetrical white matter lesions pattern which has been described in ADEM.

Currently, availability of biomarkers have been made possible not only with the diagnosis of complex entities but also understanding of their pathophysiological processes. In the case of demyelinating diseases, the presence of antibodies against myelin oligodendrocyte glycoprotein (anti-MOG) have been associated with acute disseminated encephalomyelitis, recurrent and bilateral optic neuritis and transverse myelitis in both children and adults; however, they have not been tested in the differential diagnosis of ADEM and MERS (in case they are different and not just part of a spectrum of the same disease). Additionally, the sensitivity of this antibody for a diagnosis of ADEM is as low as of 40% [19]. We did not test either anti-MOG nor aquaporine-4 antibodies in this subject since cost and availability of both tests were limiting issues, and treatment was installed immediately after admission into the hospital. Follow up showed complete resolution of both clinical and radiological features and no recurrence was seen after more than 3 years, testing both antibodies was not justified at this point of time.

A recent article that summarizes the evidence of rotavirus associated MERS found a total of 13 reported cases of MERS and showed that 10 were from Japan, one from Korea, one from China and one from Poland; the median age at presentation was 2 years (range 1–6 years) and in all cases, MERS was preceded by symptoms of gastroenteritis, such as vomiting, diarrhea and fever; Seizures (9/13; 69%) and disturbance of consciousness (8/13; 62%) were the most common neurological signs. Twelve patients (92%) had an isolated lesion in the splenium of the corpus callosum, and one patient (8%) had lesions in the splenium and genu of the corpus callosum [17]. Another study reported five cases of older age (2–26 years), all of them had sudden onset of fever and one also experienced recurrent vomiting and watery diarrhea; with respect to nervous system involvement, seizures, headache and meningeal signs were also reported. Neuroimaging showed abnormal signals restricted to the splenium of the corpus callosum (SCC) in all patients [16].

As our subject of study did not have all the clinical features (fever, gastrointestinal symptoms, seizures or meningeal signs) or the demographic characteristics of the reported cases of MERS (Asian ethnicity), it does not seem to fulfill all the criteria for that entity, besides that, our case has also imaging features not previously described for MERS such as infratentorial hyperintense images and confluent, symmetrical white matter lesions in MRI; it is important to note that the involvement of corpus callosum and restricted diffusion has also been described in the acute phase of ADEM [10] and the proposed imaging distinction between them is that in ADEM the lesions seen in the corpus callosum usually are asymmetric and contrast-enhancing [20].

Taking together the demographic, clinical and imaging features seen in our subject makes the diagnosis of ADEM and MERS overlap, a well-known condition in the scenario of post infectious encephalitis [20], in fact, the clinical and imaging spectrum of these entities is so broad that they may converge and may be seen as a single entity with a broad spectrum of manifestations (Table 1).

In this respect, and possibly related with the same spectrum of disease, tumefactive lesions (demyelinating lesions of greater than 2 cm) involving the corpus callosum can also occur in ADEM but are most closely related with MS where they often occur at the time of first presentation, either isolated or in association with other typical MS demyelinating lesions. In the largest published series of tumefactive demyelinating lesions among 168 patients, 10 patients (7%) exhibited a so-called “butterfly” appearance with symmetrical involvement of the corpus callosum and both cerebral hemispheres [21, 22].

“Transient splenial lesions” of the corpus callosum have been used to describe more restricted lesions among MERS cases, however, this term has also been employed to describe not only CNS inflammatory diseases such as Kawasaki disease, systemic lupus erythematosus and CNS infections, but also, non-inflammatory entities such as seizures (including status epilepticus), anticonvulsant use, hypoglycemia and other metabolic disturbances [21, 23].

Regarding pharmacological management, most of the published evidence for ADEM suggests high-dose corticosteroids as the first-line of treatment. Either methylprednisolone (20–30 mg/kg/day to a maximum dose of 1 g/day), or dexamethasone (1 mg/kg/day), followed by oral prednisone tapering for 4–6 weeks are the suggested schemas [24]. In the case of MERS the available literature suggest only the use of supportive measures such as hydration and antibiotics [17]. In our case, 5 days of methylprednisolone were enough to solve both clinical and radiological abnormalities. Besides this, there was no need to reinforce treatment with any other intervention, since the clinical picture completely solved after intravenous methylprednisolone was used, and clinical relapses have been negative for more than 3 years of follow-up.

The prognosis in ADEM is more severe in adults with respect to hospitalization, intensive-care unit admission, recovery and mortality [25]. The benign course and excellent response to treatment found in our patient, seems similar to children where the extent and site of lesions on initial MRI scans are not predictive of clinical outcome [26]. In general, patients with MERS type 2 lesions have also a good prognosis [20].

## 4. Conclusion

The present case showing confluent, symmetrical white matter lesions and corpus callosum involvement after an infectious disease shares clinical and radiological features of acute disseminated encephalomyelitis and mild encephalopathy with reversible splenial lesion. If MERS and ADEM are not part of the same spectrum, it is necessary to develop a biological marker that clearly distinguishes between these two entities, however due to the overlap they may be part of the same spectrum of disease.

## Figures and Tables

**Table 1:** Diagnostic criteria for ADEM, MERS type 1 and MERS type 2.

	ADEM (Krupp 2013) [4]	MERS type 1	MERS type 2 (Notebaert 2013) [20]
Clinical manifestations	(i) A first polyfocal, clinical CNS event with presumed inflammatory demyelinating cause	(i) Clinical onset associated with neuropsychiatric symptoms within 1 sweek after fever onset.	
	(ii) Encephalopathy that cannot be explained by fever	(ii) Encephalitis or encephalopathy: speech difficulties, drowsiness, decreased consciousness, delirium, seizures, irritability, agitation, and disorientation	
	(iii) No new clinical and MRI findings emerge three months or more after the onset	(iii) Complete recovery without sequelae, mostly within 10 days after the onset of neuropsychiatric symptoms	

Brain MRI	(i) Diffuse, poorly demarcated, large (>1–2 cm) lesions involving predominantly the cerebral white matter	(i) High-signal-intensity lesions on T2-weighted images, isointense to hypointense lesions on T1-weighted imaged and a homogenously reduced diffusion	(i) High-signal-intensity lesions on T2-weighted images, isointense to hypointense lesions on T1-weighted imaged and a homogenously reduced diffusion
	(ii) T1 hypointense lesions in the white matter are rare	(ii) Lesions are seen in the midline of the splenium of the corpus callosum without contrast-enhancement	(ii) Lesions affect in a variable degree the corpus callosum (from its anterior aspect to its totality) and in a bilateral and symmetrical fashion the center semiovale without contrast-enhancement
	(iii) Deep grey matter lesions (e.g. thalamus or basal ganglia) can be present	(iii) Complete resolution of the splenial lesion on repeat imaging within 1 week, with no residual signal changes or atrophy	
	(iv) Gadolinium enhancement of one or more lesions occurs in 14–30% of cases		(iii) Complete resolution of the splenial lesion on repeat imaging within 1 week, with no residual signal changes or atrophy
	(v) In addition to findings on brain MRI, patients with ADEM can have extensive lesions on spinal MRI		

ADEM = Acute disseminated encephalomyelitis, MERS = Mild encephalopathy with reversible splenial lesion, CNS = Central Nervous system, MRI = Magnetic resonance imaging.
